# Assessing the Conservation Priority of Alpine Carabid Beetle Communities by Mapping the Index of Natural Value (INV) in Natura 2000 Habitats in the Brenta Dolomites (Italian Alps)

**DOI:** 10.3390/insects16060602

**Published:** 2025-06-07

**Authors:** Emiliano Peretti, Marco Armanini, Roberta Chirichella, Andrea Mustoni, Mauro Gobbi

**Affiliations:** 1 Climate and Ecology Unit, Research and Museum Collections Office, MUSE-Science Museum, 38122 Trento, Italy; mauro.gobbi@muse.it; 2 Adamello Brenta Nature Park, Via Nazionale 12, 38080 Strembo, Italy; marcoarmanini@pnab.it (M.A.); andreamustoni@pnab.it (A.M.); 3 Department of Humanities and Social Sciences, University of Sassari, Via Roma 151, 07100 Sassari, Italy; rchirichella@uniss.it

**Keywords:** Alps, Carabidae, Coleoptera, community ecology, ground beetles, mountain ecology, priority areas, species traits, UNESCO World Heritage Site

## Abstract

The Dolomites (European Alps) are a UNESCO World Heritage Site known to harbor distinctive communities of carabid beetles adapted to high-altitude environments, whose composition is shaped mainly by landform and habitat type. We aimed to assess the conservation priority of carabid beetle communities in the Brenta mountain group (Italy). We used the Index of Natural Value (INV), based on the relative frequencies and abundances of highly specialized Alpine species, as a proxy of the sensitivity to disturbance, and, thus, to the vulnerability of the carabid beetle communities to extinction. We used information on Natura 2000 habitat cover to produce a map for identifying areas of the Brenta Dolomites inhabited by the carabid beetle communities most relevant for conservation. We also report on the main differences in terms of species composition between the investigated communities. We found a positive correlation between vulnerability and altitude. Specifically, the most vulnerable communities were recorded in high-altitude habitat types (limestone cliffs and pavements, calcareous and calcschist screes, and alpine calcareous grasslands). Alkaline fens resulted in having the least vulnerable community, while those in all other habitats (bush and forests) were found to have intermediate levels of vulnerability.

## Simple Summary

Carabid beetles are a group of insects that have been widely investigated in their relationship with environmental features of terrestrial habitats. We leveraged our knowledge on the main factors driving the species composition of carabid beetle communities in the Dolomites UNESCO World Heritage Site (Brenta mountain group, Italian Alps) to gain knowledge on which protected habitats host the most demanding and localized species. This will allow targeting conservation efforts on carabid beetle communities that are more sensitive to habitat disturbance and to climate change. We found that the most vulnerable communities inhabit rocky habitats at the highest altitudes and that, in general, vulnerability increases with altitude. We also found that habitats at the highest altitudes differ significantly in community composition compared to nearly all the other habitat types.

## 1. Introduction

The Dolomites are a well-delimited mountain range composed of carbonate platforms and buildups located in the southeastern Alps [1]. They were designated as a UNESCO World Heritage Site due to their distinctive landscape and unique geological and geomorphological features [2]. The Brenta area is the westernmost and most isolated among the nine mountain groups included in the UNESCO site (ca. 35 km far from the closest mountain group, the Corno Bianco-Weiβhorn), and it is the only one located west of the Adige Valley, in the southern Rhaetian Alps (Italy) (sensu [3]). Since 1988, it has been included in the Adamello Brenta Nature Park with the aim of protecting the Alpine and pre-Alpine ecosystems therein, as well as to promote scientific research and a sustainable use of natural resources (Trento Province Law n. 18/1988; available at https://www.consiglio.provincia.tn.it/leggi-e-archivi/codice-provinciale/Pages/legge.aspx?uid=794, accessed on 30 April 2025).

As a part of the Natura 2000 Network (SAC; i.e., Special Area of Conservation), the Brenta Dolomites include 35 habitat types with a high level of preservation, especially at the higher altitudes, which are relevant for conservation of endangered species, such as glacial relicts and alpine endemics (https://www.pnab.it/en, accessed on 16 April 2025). The Brenta Dolomites are also known to harbor a high species richness for several taxonomic groups (e.g., [4,5,6]). However, the real species diversity may be underestimated, since recent investigations resulted in the description of new species endemic to this area [7,8,9,10]. Actually, research and conservation efforts were focused primarily on vertebrate fauna [5,11,12]. In this context, the area was selected as the reintroduction site for the brown bear (*Ursus arctos*) in the Alps [13,14].

Carabid beetles (Coleoptera: Carabidae) are one of the major groups of bioindicators of environmental quality and were extensively studied in their relationship with environmental variables and habitat disturbance (e.g., [15,16,17,18]). They include several species adapted to mountain environments (e.g., [19,20]) and are one of the main groups of terrestrial arthropods in glacial and periglacial environments, both in terms of species richness and abundance [21]. In the Alpine area, they have been investigated mainly concerning their distribution patterns along altitudinal gradients (e.g., [22,23,24,25]), even in the Dolomites [26,27,28]. The distribution of carabid beetle communities in high altitude environments is driven mainly by habitat type and landform: in the Brenta Dolomites, the most specialized and ecologically demanding species were found associated to calcareous scree slopes with chasmophytic vegetation and, in particular, to high altitude landforms, such as large rockslide deposits, bedrocks, scree slopes and along glacier forelands [29]. Cold-adapted species assemblages inhabiting ice-related landform types are particularly sensitive to climate change, and a monitoring program was proposed for their conservation [30].

The aim of this work is to pinpoint the Natura 2000 habitats in the Brenta Dolomites harboring the carabid beetle communities most vulnerable to climate change and to the impacts of human activities. We also provide information on their distribution in the investigated area in relation to the current habitat cover.

## 2. Materials and Methods

### 2.1. Study Area, Sampling Activity, and Identification of the Specimens

**Figure 1:**
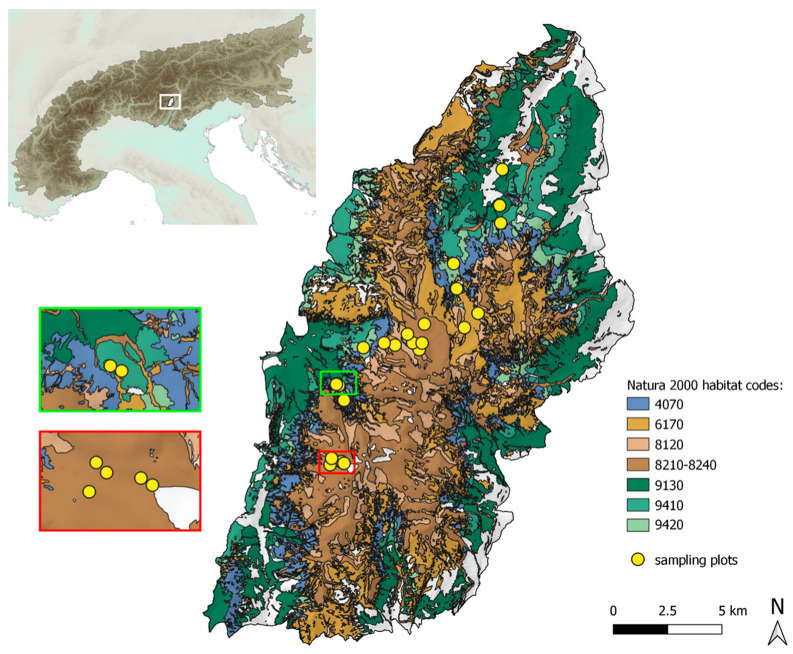
Investigated Natura 2000 habitats and sampling plots within the study area (Brenta Dolomites, within the boundaries of the Adamello Brenta Nature Park in the Alps; see top-left panel). Habitat 7230 is not shown because it covers only 1 ha in the entire study area (<1‰ of the total surface). Selected areas of the map are displayed in enhanced detail to improve readability. Cartographic data on Natura 2000 habitats are from the Geoservice of Trento Province (https://siat.provincia.tn.it/geonetwork/srv/eng/catalog.search#/home, accessed on 19 April 2025).

This study was carried out in the Brenta Dolomites (ca. 31,000 ha), Site IT3120177 of the Natura 2000 Network and part of the Dolomites UNESCO World Heritage Site, included in the Adamello Brenta Nature Park (Figure 1; https://www.pnab.it/en/).

A total of 23 linear plots, 314 m^2^ each, were investigated between 2018 and 2023 (six plots in 2018, five in 2019 and 2021, four in 2022, and three in 2023; sampling periods are given in Table S1 in the Supplementary Materials), as part of the BioMiti Project promoted by the Adamello Brenta Nature Park (https://www.pnab.it/en/research-and-biodiversity/the-biomiti-poject/, accessed on 28 April 2025). The project was aimed at developing a long-term monitoring scheme of the habitats within the Park in order to investigate the impact of climate change and habitat management on biodiversity, and to gather data to help protect the natural environment of the Park.

**Figure 2:**
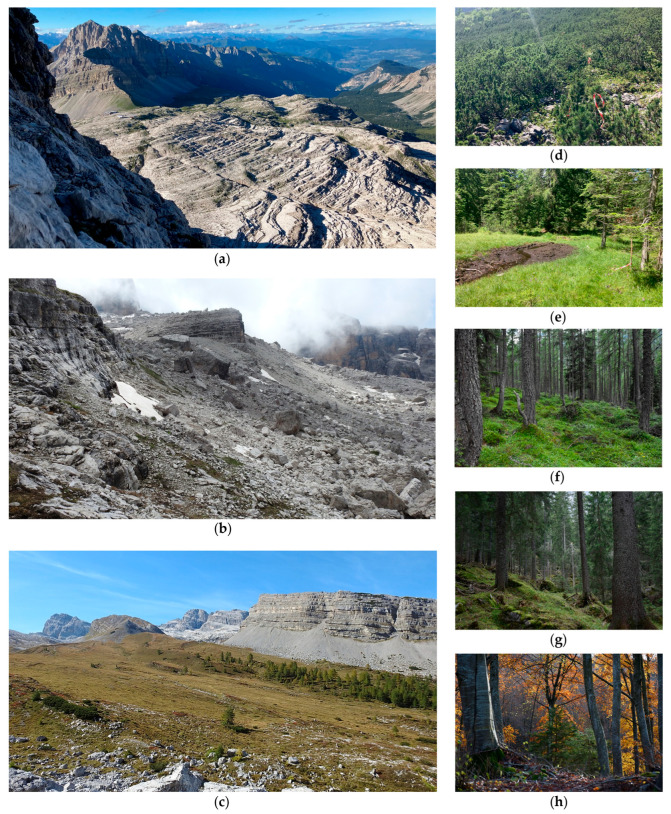
Examples of the investigated habitats, arranged approximately from the highest to the lowest altitude: (**a**) limestone cliffs with crevice vegetation and limestone pavements (habitat 8210-8240); (**b**) calcareous and calcschist screes (8120); (**c**) alpine calcareous grasslands (6170); (**d**) bushes with *Pinus mugo* and *Rhododendron hirsutum* (4070); (**e**) alkaline fens (7230); (**f**) alpine *Larix decidua* and/or *Pinus cembra* forests (9420); (**g**) acidophilous *Picea* forests (9410); (**h**) *Asperulo-Fagetum* beech forests (9130). Photos are from the archive of the Adamello Brenta Nature Park.

Within each plot, five pitfall traps were placed 50 m apart along a transect for one entire snow-free season; traps consisted of a plastic vessel (diameter 7 cm, height 10 cm) baited with a mixture of wine-vinegar and salt [29]. Traps were located between 1082 and 2891 m a.s.l. in nine different Natura 2000 habitat types (Table 1; Figure 1 and Figure 2; see also Table S1 in the Supplementary Materials). Limestone cliffs with crevice vegetation (habitat code: 8210) and limestone pavements (habitat code: 8240) are considered a single habitat unit in all subsequent analyses because many traps were placed at the boundary between the two habitats, which are known to harbor similar carabid beetle communities [29] (Figure 2a). Other investigated habitats include calcareous and calcschist screes (habitat code: 8120; Figure 2b), alpine calcareous grasslands (habitat code: 6170; Figure 2c), bushes with *Pinus mugo* and *Rhododendron hirsutum* (habitat code: 4070; Figure 2d), alkaline fens (habitat code: 7230; Figure 2e), alpine *Larix decidua* and/or *Pinus cembra* forests (habitat code: 9420; Figure 2f), acidophilous *Picea* forests (habitat code: 9410; Figure 2g), and *Asperulo-Fagetum* beech forests (habitat code: 9130; Figure 2h). Six traps were located in areas not classified as Natura 2000 habitats (Table S1 in the Supplementary Materials).

All collected carabid beetles were identified in the lab at the species level following Pesarini and Monzini [31,32], Magrini and Degiovanni [33], and Allegro [34]. Nomenclature follows Casale et al. [35]. Specimens are stored in the invertebrate miscellaneous collection of the MUSE–Science Museum in Trento (Italy).

### 2.2. Index of Natural Value and Map for Prioritization of Conservation of Carabid Beetle Communities

The Index of Natural Value (INV) proposed by Brandmayr et al. [36] (see also [37]) was used to achieve information on the distribution of the carabid beetle communities with the highest relative frequency and abundance of species with strict ecological requirements and restricted distributional range in the Brenta Dolomites.

INV was calculated for each trap on the basis of the relative frequency and abundance of brachypterous (i.e., wingless) (B), specialized zoophagous (e.g., helicophagous) (Z), and regional endemic (i.e., endemic of the European Alps) (E) species, respectively. Species exhibiting these traits are generally deemed to form highly specialized communities associated with habitats with low disturbance [25,29,38]. For each trait (either B, Z, or E), frequencies were calculated relative to the number of species in the same trap (FrT) and to the total number of species sharing the same trait in the study area (FrA). Abundance of B, Z, and E species, expressed in Activity Density (AD; i.e., number of specimens/days of activity of the trap), was calculated relative to the total AD of carabid beetles in the trap (ArT). INV was then computed as the arithmetic mean of all frequencies and abundances (i.e., FrT, FrA, and ArT of the B, Z, and E species) multiplied by 100 [39] (Table S2 in the Supplementary Materials). Traits were obtained from Chamberlain et al. [25].

We tested for correlation between the INV value and the altitude of the traps by the Kendall rank correlation coefficient to account for the non-normal distribution of the INV values and the presence of ties.

We used a Correspondence Analysis (CA; [40]) to explore the differences in the investigated traits between the carabid beetle assemblages collected by means of each trap and between the communities of the Natura 2000 habitats.

These statistical analyses were carried out in Past ver. 5.1 [41] and R ver. 4.4.3 [42].

To display the distribution of the carabid beetle communities with higher conservation priority, a map was produced by plotting the mean INV value of each Natura 2000 habitat type in the Brenta Dolomites. Maps were produced in QGIS ver. 3.24 [43].

### 2.3. Other Features of the Carabid Beetle Communities in the Natura 2000 Habitats

We tested the differences in the composition of the carabid beetle communities inhabiting different Natura 2000 habitat types by means of a one-way ANOSIM with Bray–Curtis distances and 9999 permutations [44]. We used Indicator species analysis (IndVal) to identify the species contributing significantly to the differentiation between the communities of the habitats [45]. These statistical analyses were carried out in Past ver. 5.1 [41].

## 3. Results

We collected and identified 2002 specimens belonging to 28 different species (Table 2). The species *Abax pilleri* and *Pterostichus multipunctatus* were significantly the most abundant (total AD > 10), while all the others had AD ≤ 2 (Table 2; see also Table S1 in the Supplementary Materials). *Pterostichus multipunctatus* was the only species collected in all the investigated habitats, while *Pterostichus unctulatus* was recorded in all habitats except for 8120 and 8210-8240. All the other species were recorded in ≤5 habitats; some of them were found associated with a single type of habitat (Table 2).

### 3.1. Index of Natural Value and Map for Prioritization of Conservation of Carabid Beetle Communities

Traps that did not collect any carabid beetle were not considered; therefore, the total number of traps used for the analyses is 98.

The INV value ranges 0.00–74.89 and is positively correlated with altitude (Kendall’s τ = 0.46, *p* < 0.001; Shapiro–Wilk test for normality of INV, W = 0.90, *p* < 0.001). The highest values of INV were recovered in the habitats at the highest altitudes (Table 1): limestone cliffs and pavements (8210-8240), calcschist screes (8120), and alpine calcareous grasslands (6170), where INV values ranged from 22 to 75. Habitats reaching intermediate altitudes, i.e., bushes with *Pinus mugo* and *Rhododendron hirsutum* (4070), *Picea* and *Larix decidua* and/or *Pinus cembra* forests (9410 and 9420), had a similar range of INV values, between 16 and 31. A similar range (17–30) was also recovered in *Asperulo-Fagetum* beech forests (9130) at a lower altitude (Table 1). Lastly, in alkaline fens we recorded the lowest range of INV values, i.e., from 0 to 24 (Table 1).

**Figure 3:**
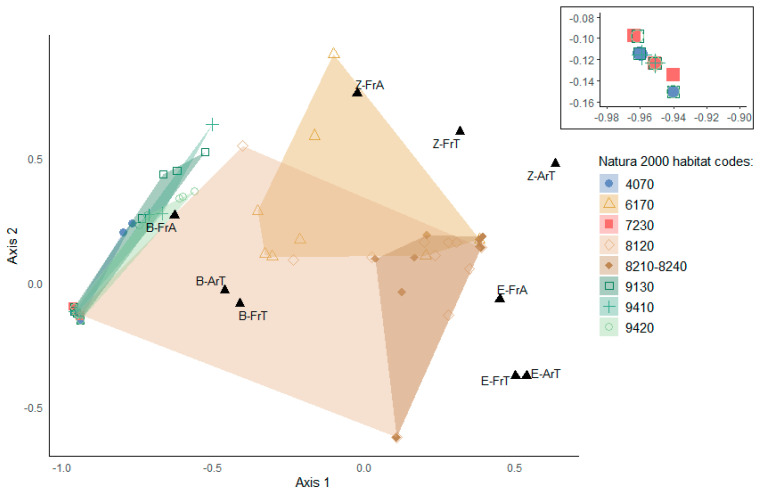
CA performed on relative frequencies and relative abundance data for each trap. Axis 1 explains 58.4% of the total variation, while axis 2 explains 28.3%. The top right panel shows a zoomed-in view of a selected area of the plot to improve readability. Abbreviations: B = brachypterous species; Z = specialized zoophagous species; E = Alpine endemic species; FrT = frequency relative to the number of species in the same trap; FrA = frequency relative to the total number of species sharing the same trait in the study area; ArT = abundance relative to the total abundance of carabid beetles in the trap.

The CA performed on the relative frequencies and abundances of B, Z, and E species produced two main axes, accounting for 58.4% (axis 1) and 28.3% (axis 2) of the total variation (Figure 3). Along axis 1, a gradient emerged between traps characterized by frequent and abundant B species, on one hand, and traps with frequent and abundant Z and E species, on the other hand. On this axis, we found a separation between traps located in bushes with *Pinus mugo* and *Rhododendron hirsutum* (4070), *Fagus*, *Picea* and *Larix decidua* and/or *Pinus cembra* forests (9130, 9410 and 9420), and alkaline fens (7230), dominated by B species and with negligible frequencies and abundance of Z and E species, and traps located in limestone cliffs and pavements (8210-8240) with both frequent and abundant Z and E species. Thus, the distribution of the traps along axis 1 may depend mainly on the arboreal vegetation cover, in correlation with altitude. Along axis 2, which separates mainly traps with higher relative frequencies and abundance of E species from traps with higher relative frequencies and abundance of Z species, we found no clear gaps between traps located in different types of habitat.

**Figure 4:**
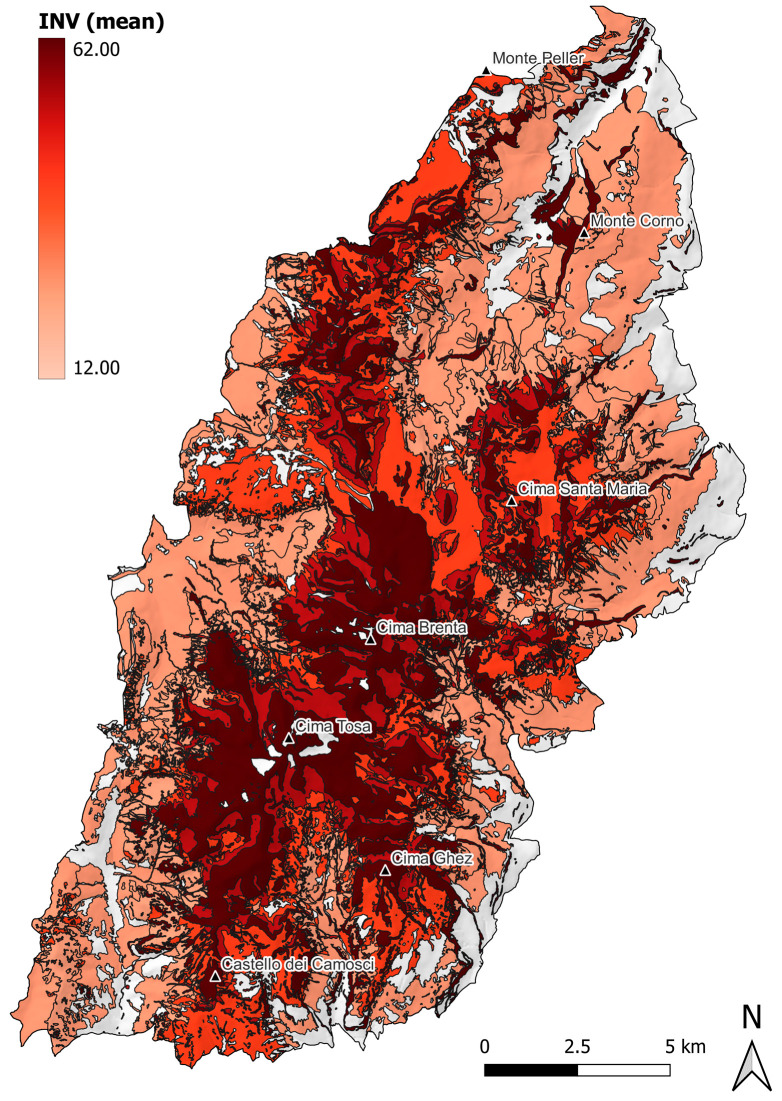
Map for prioritization of conservation of carabid beetle communities in the Brenta Dolomites (within the boundaries of the Adamello Brenta Nature Park), based on the mean values of the INV for each investigated Natura 2000 habitat type. INV values in the color scale range in an interval including the mean minimum and maximum values recovered. Key mountain summits are included to aid spatial interpretation.

A gradient of the conservation priority of the carabid beetle communities inhabiting the Brenta Dolomites within the boundaries of the Adamello Brenta Nature Park is illustrated in the map in Figure 4.

### 3.2. Other Features of the Carabid Beetle Communities in the Natura 2000 Habitats

We found the communities inhabiting the different Natura 2000 habitat types to differ significantly according to the one-way ANOSIM (R = 0.17; *p* < 0.001). In particular, significant differences emerged between habitat 8120 and habitats 4070, 9130, and 9420, between habitat 8210-8240 and habitats 4070, 7230, 9130, 9410, and 9420, and between habitat 9130 and habitat 9420 (Table 3). Five species showed a significant association with a specific type of habitat. In particular, *Pterostichus rhaeticus* is strongly associated with alkaline fens (habitat 7230; IndVal = 72.58%; *p* < 0.05, adjusted with Bonferroni’s correction), *Pterostichus unctulatus* with acidophilous *Picea* forests (9410; IndVal = 65.94%; *p* < 0.05), and *Abax pilleri* with *Asperulo-Fagetum* beech forests (9130; IndVal = 57.67%; *p* < 0.05). A less strong but significant association also emerged between *Calathus melanocephalus* and calcareous grasslands (6170; IndVal = 36.36%, *p* < 0.05), and between *Carabus adamellicola* and limestone cliffs with crevice vegetation and limestone pavements (8210-8240; IndVal = 24.75%; *p* < 0.05).

## 4. Discussion

The findings of this study show that the conservation priority of carabid beetle assemblages changes in relation to the habitat type and along the elevational gradient. Additionally, we found that Natura 2000 habitat types have a different relevance in supporting ground beetle communities with different extinction risks.

The application of the INV allowed us to identify species assemblages that are particularly sensitive to climate change and habitat disturbance. High altitude habitats, such as calcareous and calcschist screes, and limestone cliffs and pavements (8120 and 8210-8240) together occupy ca. 30% of the investigated study area (Table 1; Figure 1). They turned out to harbor carabid beetle communities with comparatively high conservation priority (Figure 4), mainly due to the relatively high frequencies and abundances of Alpine endemic and specialized predator species (Figure 3). Noteworthy, these communities are characterized by the occurrence and abundance of *Carabus adamellicola*, a stenoendemic species of the Adamello-Presanella and Brenta mountain groups. Moreover, high-altitude habitats are inhabited by *Nebria germarii*, a species that warrants conservation concern, since reductions in its distribution range, local extinctions, and its association with glacial environments have already been documented within the Park’s territory [20].

Alpine calcareous grasslands (6170) occupy around 18% of the study area (Table 1; Figure 1). According to the literature, alpine grasslands are inhabited by communities of carabid beetles with high species richness and in which functional traits (e.g., feeding habits and dispersal abilities [23]) are known to be affected by management methods, such as mowing and grazing [46,47], and threatened by upward shifts in tree-line vegetation [48].

Bush and forest habitats (4070, 9130, 9410, and 9420) are mostly contiguous within the study area and, taken together, occupy ca. 38% of its total surface (Table 1; Figure 1). Vulnerability is mainly driven by the presence of low dispersal (i.e., brachypterous) species (Figure 3). Among all the investigated habitat types, bushes with *Pinus mugo* and *Rhododendron hirsutum* (4070) is the only one listed as priority habitat in danger of disappearance [49]. However, the carabid beetle community was found to be among the least vulnerable, mainly due to the low frequency and abundance of specialized zoophagous species and the absence of Alpine endemic species (Figure 3 and Figure 4; Table 2; see also Table S1 in the Supplementary Materials). As already pointed out by Pizzolotto [28], this type of habitat shares its carabid beetle community with *Larix decidua* forests.

The relatively low vulnerability we recovered for the carabid beetle community inhabiting the alkaline fens (7230; Table 1) is probably due to the groundwater level fluctuations. The limited sampling effort employed in this habitat (n of traps = 4; see Table S1 in the Supplementary Materials) is justified by the very small area covered by it (<1‰ of the total surface); moreover, we aimed at reducing the risk of capturing protected vertebrates like *Rana temporaria*, *Zootoca vivipara,* and small mammals [50].

The map in Figure 4 provides a visual estimate of the importance of different areas in the Brenta Dolomites in the conservation of carabid communities (cfr. [36]). The positive correlation between INV values and altitude confirms that high-altitude species assemblages deserve priority in conservation planning [30]. High-altitude habitats are not only the most threatened by climate change, but they also harbor the ground beetle communities that are most specialized to live in such environments and therefore most susceptible to global warming [30,51,52]. In particular, warming and drying in mountain areas may trigger habitat shift, and this is expected to affect the distribution of species with specific traits, like specialized predators [53].

The environments investigated in this research are widespread across the southeastern Alps [28]. Within the study area, they are encompassed by several conservation designations, including the UNESCO World Heritage Site, the Natura 2000 Habitat Network, and the Adamello Brenta Nature Park; this may prevent potential impacts from human activities, such as expansion of existing ski areas [54,55] and overtourism [56]. However, the relevance of these environments in the conservation of communities of highly specialized, soil-related animals, such as carabid beetles, has rarely been advocated and, as far as we know, never properly investigated.

Ecological indices are often used to inform management strategies and decisions, as well as communication tools, since they collapse information from multiple indicators into a single value [57,58]. However, they have often been criticized for potentially oversimplifying complex systems, which may lead to misinterpretation of underlying ecological processes [58]. In the context of Alpine carabid beetle communities, the ecological characteristics and responses to environmental pressures have been extensively studied in the past, often using the same functional traits applied here [25,26,27,28,29]. As such, we consider the INV a reliable proxy for gauging the sensitivity of these communities to perturbations and, consequently, their vulnerability to extinction. Instead, diversity metrics not based on traits, like species richness (and, similarly, evenness), may be misleading because they do not always correlate with environmental features [59,60]. Moreover, the index demonstrated practical value as a conservation prioritization tool: it is more straightforward and easier to interpret than relying on multiple indicators, thereby enhancing its applicability in conservation planning by managers of protected areas.

The application of the INV for the first time within protected areas and sites of global significance (e.g., UNESCO Sites) opens up new opportunities for investigating and enhancing the naturalistic and biogeographic role of these areas. In addition, it offers the opportunity to obtain a synthesis tool useful for identifying priority areas from a conservation perspective that require appropriate protection measures.

## Figures and Tables

**Table 1:** Approximated area, altitudinal range, and INV range values for each Natura 2000 habitat type. Details about each individual trap are given in Table S1 in the Supplementary Materials.

Habitat Code	Area (ha)	Altitude of Traps (m a.s.l., min.–max.)	INV (min.–max.)
4070	2500	1668–2143	16.50–29.15
6170	5600	2047–2310	21.51–74.89
7230	1	1652–1654	0.00–23.53
8120	3100	2069–2685	23.53–72.47
8210-8240	6200	1697–2891	45.64–74.89
9130	5500	1309–1502	17.04–29.71
9410	2300	1651–1653	15.93–30.39
9420	1400	1858–1943	22.88–31.21

**Table 2:** List of collected species, their total abundance (in AD), and presence/absence within each Natura 2000 habitat type (for details, see Table S1 in the Supplementary Materials).

Species	Total AD	Presence (+) or Absence (−) in Habitats							
		4070	6170	7230	8120	8210-8240	9130	9410	9420
*Abax parallelepipedus* (Piller & Mitterpacher, 1783)	2.23	−	−	+	−	−	+	−	−
*Abax pilleri* Csiki, 1916	11.17	+	−	−	+	−	+	−	+
*Amara quenseli* (Schönherr, 1806)	0.01	+	−	−	−	−	−	−	−
*Bembidion bipunctatum* (Linnaeus, 1761)	0.02	−	−	−	−	+	−	−	−
*Bembidion glaciale* Heer, 1837	0.02	−	−	−	−	+	−	−	−
*Calathus fuscipes* (Goeze, 1777)	0.01	−	−	−	−	+	−	−	−
*Calathus melanocephalus* (Linné, 1758)	0.09	−	+	−	−	−	−	−	−
*Calathus micropterus* (Duftschmid, 1812)	0.19	+	−	−	−	−	+	+	+
*Carabus adamellicola* Ganglbauer, 1904	2.01	−	+	−	+	+	−	−	−
*Carabus convexus* Fabricius, 1775	0.05	−	+	−	−	−	−	−	−
*Carabus creutzeri* Fabricius, 1801	0.64	−	+	−	+	+	−	−	−
*Carabus depressus* Bonelli, 1810	0.06	−	−	−	−	−	−	+	−
*Cychrus angustatus* Hoppe & Hornschuch, 1825	0.01	−	−	−	−	−	−	−	+
*Cychrus attenuatus* (Fabricius, 1792)	0.15	+	+	−	−	−	+	+	+
*Cychrus italicus* Bonelli, 1810	0.01	−	−	−	−	−	−	−	−
*Cymindis vaporariorum* (Linné, 1758)	0.08	+	+	−	−	+	−	−	−
*Duvalius nambinensis* Boldori, 1935	0.02	−	−	−	−	+	−	−	−
*Harpalus latus* (Linné, 1758)	0.06	−	+	−	+	−	−	−	−
*Harpalus solitaris* Dejean, 1829	0.05	−	+	−	−	−	−	−	−
*Leistus nitidus* (Duftschmid, 1812)	0.21	−	−	−	−	−	+	+	+
*Nebria castanea* (Bonelli, 1810)	0.12	+	−	−	+	+	−	−	+
*Nebria germarii* Heer, 1837	1.95	−	−	−	+	+	−	−	−
*Notiophilus biguttatus* (Fabricius, 1779)	0.09	+	−	−	−	+	+	+	−
*Pterostichus burmeisteri* Heer, 1838	0.91	−	−	−	−	−	+	−	−
*Pterostichus multipunctatus* (Dejean, 1828)	14.63	+	+	+	+	+	+	+	+
*Pterostichus rhaeticus* Heer, 1837	0.22	−	−	+	−	−	−	+	−
*Pterostichus unctulatus* (Duftschmid, 1812)	0.99	+	+	+	−	−	+	+	+
*Trechus sinuatus* Schaum, 1860	0.02	−	−	−	+	−	−	−	−

**Table 3:** Pairwise ANOSIMs (R) between carabid beetle communities of the Natura 2000 habitats. Statistical significance is indicated by asterisks, where * *p* < 0.05, and ** *p* < 0.01 (*p*-values adjusted with Bonferroni’s correction).

Habitat Code	6170	7230	8120	8210-8240	9130	9410	9420
4070	0.01	0.10	0.24 *	0.23 **	0.20	0.06	0.17
6170		0.15	0.08	0.06	0.30	0.11	0.14
7230			0.23	0.33 **	0.35	0.43	0.47
8120				0.03	0.29 *	0.26	0.35 *
8210-8240					0.35 **	0.33 **	0.35 **
9130						0.32	0.44 *
9410							0.11

## Data Availability

The dataset used in this paper is publicly available in the Supplementary Materials.

## Supplementary Materials

The following supporting information can be downloaded at: https://www.mdpi.com/article/10.3390/insects16060602/s1, Table S1: Natura 2000 habitat code, altitude, period of activity, and activity density (AD) of the species for each trap. For each species, we indicate the presence of the following traits: brachyptery (B), specialized zoophagy (Z), and Alpine endemism (E); Table S2: Formulas used for the calculation of relative frequencies and abundances of brachypterous, Alpine endemic, and specialized zoophagous species and of INV. In the formula used to calculate the INV, the denominator corresponds to the number of investigated parameters (in this study, n = 9).
