# Supplementary material for: Assessing the Conservation Priority of Alpine Carabid Beetle Communities by Mapping the Index of Natural Value (INV) in Natura 2000 Habitats in the Brenta Dolomites (Italian Alps)
Source: Insects. 2025 Jun 7;16(6):602. doi: 10.3390/insects16060602 (PMC12193764; doi:10.3390/insects16060602)
Supplement: Supplementary file 1 [file insects-16-00602-s001.zip › Peretti_et_al._Table_S2.pdf]

Table S2. Formulas used for the calculation of relative frequencies and abundances of brachypterous, Alpine endemic, and specialized zoophagous species and of INV. In the formula used to calculate the INV, the denominator corresponds to the number of investigated parameters (in this study, n = 9).

Parameter	Formula
B-FrT	n. of brachypterous species / n. of species in the trap
B-FrA	n. of brachypterous species / n. of brachypterous species in the study area
B-ArT	DA of brachypterous species / total DA of carabid beetles in the trap
E-FrT	n. of Alpine endemic species / n. of species in the trap
E-FrA	n. of Alpine endemic species / n. of Alpine endemic species in the study area
E-ArT	DA of Alpine endemic species / total DA of carabid beetles in the trap
Z-FrT	n. of specialized zoophagous species / n. of species in the trap
Z-FrA	n. of specialized zoophagous species / n. of specialized zoophagous species in the study area
Z-ArT	DA of specialized zoophagous species / total DA of carabid beetles in the trap
**INV**	$= \frac{\textbf{B-FrT + B-FrA + B-ArT + E-FrT + E-FrA + E-ArT + Z-FrT + Z-FrA + Z-ArT}}{9} \times 100$
